# Patient satisfaction after total knee replacement—still a challenge

**DOI:** 10.1080/17453674.2020.1763581

**Published:** 2020-05-13

**Authors:** Jan Verhaar

**Affiliations:** Department of Orthopaedics and Sports Medicine, Erasmus University Medical Center Rotterdam, the Netherlands

In the 1990s patients reported outcome measures (PROMS) were developed to reduce the risk of bias if outcome is rated by the surgeon. When the Swedish Knee Registry sent out a mail in 1999 to validate their registry to check the revision status of the patients, they included a simple question “How satisfied are you with your knee replacement?” 95% of all patients responded and were clearly less positive than expected. Robertsson et al. (2000) reported that 17% of total knee replacement (TKR) patients were either dissatisfied or uncertain with respect to the outcome. This is lower than satisfaction after total hips replacement (THR). Since this key publication, the rate of satisfaction has been studied in many other groups of patients and found to be consistent in many countries. Only 4 out of 5 patients are satisfied after TKR (Bourne et al. 2010, Dunbar et al. 2013, Bryan et al. 2018).

Identifying the causes of dissatisfaction is important in order to improve patient selection for TKR, adjust treatment strategies and to support or treat dissatisfied patients with their residual complaints. Sociodemographic, preoperative, operative, and postoperative factors have been studied in large reviews. No specific single leading factor has been found, but patients expectations, higher function before surgery, lower stage of arthritic disease, complications, poor resolution of pain, and lower improvement of knee function were more common in dissatisfied patients (Gunaratne et al. 2017). Patients with a better preoperative mental function were more often satisfied (Vissers et al. 2010, 2012). However, in almost all studies it was found that unfulfilled expectations were the main reason for dissatisfaction. Many studies advised improving patient information and education preoperatively (Conner-Spady et al. 2020, Ghomrawi et al. 2020). Tilbury et al. (2016) reported that in dissatisfied patients unfulfilled expectations were found for “improvement walking ability middle long distances” (40%), “being able to kneel down” (47%) and “being able to squat”(44%).

20 years ago in *Acta Orthopaedica* Robertsson’ s publication (Robertsson et al. 2000), opened the eyes of orthopedic world: there was a discrepancy between patient and surgeon satisfaction after TKR. Unmet expectations are a main source of patient dissatisfaction and patients have the right to be informed about the limitations that current replacement techniques have. Over the past 2 decades, new knee implants have been introduced as well as new techniques including; computer assisted surgery, patients specific guides and alternative alignment techniques. However, in unbiased studies none of these techniques and implants have shown a significant improvement of patient satisfaction.

The gap between the satisfaction rates of THR and TKR may be caused by the more complex nature of the knee joint compared to the hip. The anatomy of the knee ligaments and the individual form and size of femur, tibia and patella may be better addressed with a customized patient specific prosthesis implanted with a surgical robot to optimize precision (Namin et al. 2019, Robinson et al. 2019). Both developments are underway and may lead to a paradigm shift in TKR necessary to overcome the high percentage of dissatisfied patients. It is very important to analyze patients experiences when introducing these techniques. Based on the expected considerable increase of costs of the TKR procedure health economics also need to be studied.

Until real improvements are achieved, we orthopedic surgeons should be humble and realistic. TKR is a good, but not ideal, option for patients with significant complaints due to end-stage arthritis. We need to be careful in young patients, those with unbearable pain for which narcotics are used, and patients who want to resume high level sports activities. Reduction of pain and improvement of function may be expected but some complaints may persist. There are also possible complications including infection and thrombosis, which occur in less than 5% of patients, but may create more problems than preoperatively.

Pain relief and improving physical function are the main aims of TKR. Expectations should be explicitly addressed before surgery; a lesson now 20 years old, yet still true today.

Jan Verhaar *Department of Orthopaedics and Sports Medicine,* *Erasmus University Medical Center Rotterdam,* *the Netherlands* *E-mail:*j.verhaar@erasmusmc.nl
